# Reducing drinking in concurrent problem alcohol and illicit drug users: an impact story

**DOI:** 10.1186/1745-6215-16-S3-P11

**Published:** 2015-11-24

**Authors:** Jan Klimas, Helen Tobin, Catherine-Anne Field, Clodagh SM O'Gorman, Liam G Glynn, Eamon Keenan, Jean Saunders, Gerard Bury, Colum Dunne, Walter Cullen

**Affiliations:** 1 British Columbia Centre for Excellence in HIV/AIDS, St. Paul’s Hospital, Vancouver, Canada; 2 School of Medicine and Medical Science, University College Dublin, Dublin, Ireland; 3 Health Promotion School of Health Sciences, National University of Ireland, Galway, Ireland; 4 Graduate Entry Medical School, Faculty of Education and Health Sciences, University of Limerick, Limerick, Ireland; 5 Department of Paediatrics, Mid-Western Regional Hospital, Limerick, Ireland; 6 Department of General Practice, National University of Ireland, Galway, Ireland; 7 Addiction Services, Health Service Executive, Dublin, Ireland

## Background

One out of three people who receive methadone in primary care drink in excess of the recommended limits. This poses significant risk to their health, especially to their liver; it complicates their care and increases risk of relapse.

## Objective

To inform addiction treatment in primary care with respect to psychosocial interventions to reduce drinking in concurrent problem alcohol and illicit drug users, by: exploring the experience of (and evidence for) psychosocial interventions, developing and evaluating a complex intervention to improve implementation. Evaluation of the intervention tested core feasibility and acceptability outcomes for patients and providers.

## Methods

A Cochrane review found only four studies. Having inconclusive evidence, we interviewed 28 patients, 38 physicians and nurses. Patient interviews informed development of a national clinical practice guideline, as well as design and outcomes of the evaluation project. Feasibility outcome measures included recruitment, retention, completion and follow-up rates, as well as satisfaction with the intervention. Secondary outcome was proportion of patients with problem alcohol use at the follow up, as measured by Alcohol Use Disorders Identification Test.

## Results

Information from the Cochrane review and the qualitative interviews informed an expert panel consultation which developed clinical guidelines for primary care.

## Conclusions

The guideline became part of a complex intervention to support the uptake of psychosocial interventions by family physicians; the intervention is currently evaluated in a pilot controlled trial. Two new alcohol education programmes were created as a response of the community to the problem and a lack of specialist support services for patients with dual dependencies. Both Coolmine Therapeutic Community and the Community Response Agency run a 10-week group that specifically seeks to include people with dual dependencies, from methadone programmes.

